# Nurses’ discursive construction of older adult immigrant patients in hospitals

**DOI:** 10.1186/s12913-023-09590-6

**Published:** 2023-06-07

**Authors:** Lisbeth Alnes Vestgarden, Elisabeth Dahlborg, Jeanne Strunck, Elin Margrethe Aasen

**Affiliations:** 1 Department of Health Sciences in Aalesund, Faculty of Medicine and Health Sciences, Norwegian University of Science and Technology (NTNU), Box 1517, Aalesund, 6025 Norway; 2 Department of Health Sciences, University West, Trollhättan, 46132 Sweden; 3 Department of Culture and Learning, Faculty of Social Sciences and Humanities, Aalborg University, Kroghstraede 3, Aalborg, 9220 Denmark

**Keywords:** Nurses, Nurse-patient relations, Older adult immigrants, Qualitative research, Critical discourse analysis, Patient constructions

## Abstract

**Background:**

The immigrant population across Europe is ageing rapidly. Nurses will likely encounter an increasing number of patients who are older adult immigrants. Moreover, access to and equal provision of healthcare is a key issue for several European countries. The relationship between nurses and patients is asymmetrical with unequal power relations; however, the way nurses construct the patient through language and discourse can help maintain or change the balance of power. Unequal power relations can affect access and be a hindrance to equal healthcare delivery. Hence, the aim of this study is to explore how older adult immigrants are discursively constructed as patients by nurses.

**Methods:**

An exploratory qualitative design was used. Data were collected through in-depth interviews with a purposive sample of eight nurses from two hospitals. The nurses’ narratives were analysed using critical discourse analysis (CDA) as described by Fairclough.

**Results:**

The analysis identified an overarching, stable, and dominant discursive practice; ‘The discourse of the other’, with three interdiscursive practices: (1) ‘The discourse on the immigrant patient versus an ideal patient’; (2) ‘The expert discourse’; and (3) ‘The discourse of adaption’. Older immigrant adults were constructed as ‘othered’ patients, they were different, alienated, and ‘they’ were not like ‘us’.

**Conclusion:**

The way nurses construct older adult immigrants as patients can be an obstacle to equitable health care. The discursive practice indicates a social practice in which paternalism overrides the patient’s autonomy and generalization is more prevalent than a person-centred approach. Furthermore, the discursive practice points to a social practice wherein the nurses’ norms form the basis for normal; normality is presumed and desirable. Older adult immigrants do not conform to these norms; hence, they are constructed as ‘othered’, have limited agency, and may be considered rather powerless as patients. However, there are some examples of negotiated power relations where more power is transferred to the patient. The discourse of adaptation refers to a social practice in which nurses challenge their own existing norms to best adapt a caring relationship to the patient’s wishes.

**Supplementary Information:**

The online version contains supplementary material available at 10.1186/s12913-023-09590-6.

## Background

### Introduction

Worldwide, many people live in a country outside their country of birth. Europe is the region that hosts the largest number of international immigrants; in 2020 this amounted to 87 million people, of whom about 40 million are non-European [1]. In the Nordic countries, the number of immigrants varies: Finland has the lowest number (7.4%), Sweden the highest (19.7%), and Norway is in the middle (16.3%) [2].

The immigrant population across Europe is aging, but significant differences exist between countries in terms of age composition [3]. Most immigrants who arrived in north-western Europe between the late 1950s and the mid-1970s were young adults seeking post-war work. Many have since returned to their home countries; however, a significant number have remained and lived in Europe for decades [4]. These immigrants are of retirement age or older and need access to health care in their country of residence. In Norway, the number of older immigrant adults is projected to increase sharply in the coming years. In 2022, immigrants accounted for 7% of the population aged 60 and over, but this is expected to increase to 24% by 2060 [5]. Today, most older immigrants in Norway are from European countries (60% in 2022), but forecasts indicate that this will change; in 2060, most older immigrants in Norway will have a background from countries outside Europe [5, 6].

This paper focuses on immigrants from countries outside Europe who are aged 65 years and above, and how nurses talk about them as patients in hospitals.

### Access to healthcare for immigrants

Health is considered a human right; however, there are differences in access and the way health services are organized in European countries, both for immigrants and the majority population [3]. A review by Lebano et al. [7] examined access to healthcare in a European context, and found inequalities between immigrants and non-migrants, despite the aspiration to ensure equality of access. At the same time, immigrants are considered to be at greater risk of health problems for reasons that include the migration process and the consequences of economic and social marginalization [3].

Though immigrants in Norway have the same legal rights to health and care services as the rest of the population, they use the services somewhat differently. Previous studies have revealed that older immigrant adults in Norway do not consult primary health services as often as the majority population does [8]. Furthermore, they are admitted to somatic hospitals less frequently [9] and use the psychiatric specialist health service less frequently compared to the majority population [10], despite having higher rates of mental health concerns than the rest of the population [11].

Differences in the use of healthcare services for older immigrants may be attributable to different access barriers [12]. Care providers are often unaware of these barriers, although they can be found at the patient, provider, and system levels [13]. Immigrants experience multiple barriers to seeking and receiving healthcare services, such as language skills, lower health literacy, different health beliefs, and uncertainty about the role of health personnel [14]. A lack of knowledge about the health service, and how to navigate the healthcare system can be a barrier as well because information is rarely available in languages ​​other than the host language and English [15, 16]. A study conducted in Norway revealed that immigrants who were not proficient in the Norwegian language had significantly poorer levels of health than those with better proficiency [17]; hence, the inability to speak the host country’s official language is more than a communication barrier, and language facilitation must be seen as a structural factor for gaining access to health services [18]. Barriers also exist at the provider level and include communication behaviour and preconceived stereotypical attitudes among professionals [3, 13]. In addition, a review from the US points to the need for stronger interpersonal relationships and better-prepared staff [19]. Healthcare professionals may lack sufficient cultural competence, training, and education [20]. Experiences of racism and discrimination are also barriers [21]; immigrants in Norway have felt ignored and treated as second-class citizens by health professionals, which negatively affects future health patterns [16]. The relationship between healthcare personnel and patients, in general, is characterized by an uneven distribution of power [22]; healthcare personnel have expert and institutional knowledge, and this puts patients in a more vulnerable position. Bradby [22] highlighted how this vulnerability makes it harder for patients to resist structural inequalities where ‘abnormal’ conditions, such as racism, appear ‘normal’ and invisible. Furthermore, a study by Arora et al. [18] highlighted how public discourses contributed to ethnic boundary-making in healthcare interactions, thus creating access barriers to healthcare services for Pakistani women. The study also pointed to how the intersection between ethnicity, age, gender, and social roles affected power structures and inequality in access to health services [18]. In a Nordic context, Debesay et al. [23] revealed that immigrants, especially older immigrant women, have less access to healthcare services, and this seems to be connected with their lower socioeconomic status. However, despite several studies in recent years, knowledge about access and health barriers for older immigrants in Europe is still fragmented and insufficient [12, 24].

### The changing role of the patient

Traditionally, healthcare service has been dominated by a paternalistic ideology. Paternalism refers to intentionally overriding another individual’s preferences or actions and, therefore, contrasts with individual autonomy characterized by one’s freedom to act in accordance with a self-chosen plan [25]. Hence, healthcare professionals have assumed an expert role, while the patient is a passive recipient. During the post-war era, the sociologist Talcott Parsons characterized the ‘sick role’ and elucidated the rights and responsibilities of those who are sick. The sick person was expected to seek competent help and recover by following professional advice [26]. Parsons’ sick role, which was accepted until about 1990, was criticized because, among other things, the sick person was expected to accept their diagnosis and follow the doctor’s recommendations without protest [27].

In recent decades, there has been widespread acceptance, in political and policy declarations, that patients should be more involved in care, the treatment process, and decision-making [28]. The common goal is that patients should be treated as persons and that health services should be rooted in universal principles of human rights and dignity, non-discrimination, participation, and partnership of equals. Furthermore, care should consider the person’s needs, wants, and preferences [28]. In Europe, the Nordic countries were among the first to formalize patient rights [29]; patient participation is a statutory right in several countries, including Norway, Sweden, and Denmark [30–32]. The legislation states that healthcare services must be designed in collaboration with the patient, as far as possible, and that the patient’s opinions must be considered. However, even though patient participation has been an explicit health policy ideal in recent years, analyses of key health policy documents in three Nordic countries revealed that patients are still constructed as passive, with limited authority to participate [33–35]. Similarly, Dahl et al. [36] revealed that in Nordic governmental policy documents (not limited to the healthcare context), immigrants were constructed as passive service recipients with little agency or influence and were seen as a problem rather than a resource for society.

### Language and discourses

Critical discourse analysis (CDA), which forms this study’s theoretical background, is used to study how language creates meaning and informs social practice [37]. Discourse is a way of representing certain aspects of the material, mental, or social world, and CDA emphasizes that although discourse is language, it is also a form of social practice rather than an individual activity [37]. CDA is concerned with how power is created and exercised through language, as there is power *in* and *behind* discourses. Thus, language can lead to the domination of some people over others [38].

Language is essential for nurse-patient interactions, as discourses construct our perceptions of relationships, identity, knowledge, and power [37]. Discourses also form part of what people use when relating to something important to them [38]. For example, the way nurses talk and express themselves is not a neutral reflection of their context or social relations. Conversely, the way nurses express themselves is based on an accepted approach of conducting conversations in the specific healthcare context and the discourse they choose to lean on, such as medical, legal, or a more regular discourse. The nurse positions patients as social subjects by adopting different discourses, which leads to the construction of social identity and social relationships. Regardless of the choice of discourse, the nurses’ relationship with their surroundings and to hierarchies of power is affected [37]. Prevailing norms are one of the factors influencing the way nurses construct patients. Norms regulate human ideas, identity, and conduct, and govern the desirable, normal, or abnormal in a society. Norms are invariably constructed in relation to something that does not fit the norm [39].

Discourses are essential in the asymmetric nurse-patient relationship, as the nurses’ construct of the patient can help maintain or change the unequal balance of power [38]. Unequal power relations can have major ideological effects [37] and, thus, influence care on equal conditions [40]. Hence, the language and discourses used by nurses may hinder healthcare delivery and equal care. A critical investigation of the nurses’ language can illuminate their use of discourses and reveal a characterization of power and dominance and, thus, the expression of power in the caring relationship.

Although communication behaviour among professionals is referred to as a healthcare barrier [3], there is still, as far as we know, no exploration in Norway or globally of the language and discourses used by nurses to construct older adult immigrants as patients.

## Methods

### Aim and objective

This study aims to explore how older adult immigrants are discursively constructed as patients by nurses.

### Design

An exploratory qualitative study was employed, based on nurses’ narratives of older immigrant patients. CDA was applied as a theoretical framework and a method of analysis [37]. CDA is based on the idea that the discourses used by the nurses will influence and be influenced by social practices. This is a social constructivist approach, wherein reality is understood as socially constructed, and ‘truth’ is discursively produced. Discourses are constructed by and frame reality and reality, in turn, constructs discourses [41].

### Population and sample

Purposive sampling [42] was used to obtain data on the nurses’ construct of older immigrant patients. Specific inclusion criteria were selected to obtain the most information-rich participants possible: (i) nurses currently employed at a hospital; (ii) minimum of two years’ length of service; and (iii) experience with immigrant patients (aged 65+) from countries outside Europe. Forecasts estimate that most of the future older immigrants in Norway will be born in countries outside Europe [5]; hence, we chose nurses who had experience with these patients.

Four hospitals in Norway were invited to participate in the study; two of them accepted. Both of these hospitals offer emergency care for approximately 200 000 inhabitants and are located in cities with a large immigrant population. One medical ward in each hospital was chosen to participate by the respective heads of research. The nurses were notified about the project and asked to participate by the charge nurse in each ward. The charge nurses were informed that variation and diversity in age, work experience, ethnicity, and gender were desirable. In total, eight participants were recruited (Table 1); no information is available on the number of nurses who refused to participate. All participants met the inclusion criteria. Although the participants reflect variation in age and work experience, only women and ethnic Norwegians were recruited. This probably reflects that most employees were ethnic Norwegian women. Based on the contact information shared by the charge nurse, the first author scheduled appointments and conducted the interviews.

**Table 1:** Participants

Gender	8 women	0 men
Age (Average: 40 years)	25–36 years (N1, N2, N5, N7) 42–63 years (N3, N4, N6, N8)	
Work experience as a nurse (Average: 7 years)	2–8 years (N1, N2, N5, N6, N7) 13–20 years (N3, N4, N8)	

N: Nurse

### Data abstraction/collection

The study data were collected via eight individual face-to-face interviews using open-ended questions in a narrative interview format. The narrative form of communication is appropriate for organizing events, actions, feelings, or thoughts regarding others [43]. The interviews were conducted from January 2020 to March 2020; five were conducted during the nurses’ working hours, and three during their leisure hours. All interviews were conducted in the hospitals’ meeting rooms, except one, which was conducted at a café. The nurses were informed in advance by verbal and written communication that the interview topic would revolve around their experiences with older adult immigrant patients who were born and raised in countries outside Europe. The interviews began with the researcher introducing herself as an academic nurse educator and former clinical nurse. This was followed by a self-introduction by the nurses; subsequently, they were asked to share their experiences with immigrant patients. There is limited knowledge about nurse-patient interaction when the patient is an older adult immigrant; therefore, the objective was to conduct an open and receptive dialogue to enable the informant to speak as freely as possible. The first author, who conducted all the interviews, encouraged the nurses to narrate specific accounts and requested further details by asking questions, such as ‘Could you tell me more about that situation?’; ‘What did the patient say/do then?’; or ‘What did you say/do then?’. The interviews were audio recorded and subsequently transcribed verbatim by the first author. Each interview lasted 40–60 min, with the average length being 51 min.

### Data analysis

The nurses’ narratives were analysed using CDA and Fairclough’s three-dimensional model of discourse analysis [37, 38]. This model explores the link between text/language and social practice, in the following three dimensions: (1) ***description*** of text; (2) ***interpretation*** of discursive practice, and (3) ***explanation*** of the social practice. Prior to the analysis, the first author listened to the audio recordings and then transcribed them. All interviews were then read thoroughly and repeatedly, to receive an impression of the content of the text. Paragraphs and sentences wherein the nurses spoke about older adult immigrants were subsequently selected for analysis. Examples of selected sentences are: ‘They can be tiresome; they call a lot and ask about minor matters’ (Nurse 1), and ‘It makes it a bit wrong that they… when I think that you should be able to do this’ (Nurse 8). The NVIVO software was used to keep track of the selected sections, and the subsequent text descriptions.

#### Description of the text (The first dimension of the analysis)

This was a detailed linguistic examination of the selected text, focusing on the words employed [37]. The following queries were used: What words and personal pronouns did the nurses use to refer to immigrant patients and themselves? Did they choose value-laden words, such as frustrating, or resourceful? Which metaphors or other linguistic images did they employ? Did the text contain words involving an element of assessment, such as often, never, everybody, or nobody? Did the nurses use modal auxiliary verbs, such as must, should, or will?

Grammatical level expresses a range of modalities [37] yielding differing inputs on the level of power in the patient-nurse relationship and the positioning of the nurse relative to the patient. Metaphors are relevant because human thought processes are metaphorical; metaphors structure our knowledge and belief systems and how we think and act [37].

#### Interpretation of discursive practice (The second dimension of the analysis)

Interdiscursivity is the expression and combination of different types of discourse in the text [37]; in this study, the text type or genre was the narrative interviews between the researcher and nurses. In the second dimension, all authors discussed and interpreted the discursive practice in the text based on the features, patterns, and structures outlined from the linguistic analysis in the first dimension. Furthermore, the interpretation was influenced by the study objective as well as the authors’ professional background and (pre)-understanding (three academic nurse educators and one linguist). The process was repeated several times, and the authors’ interpretation was as follows: four discourses, as described in the Results section; one discourse was overarching, dominant, and stable, with three interdiscursive practices.

#### Explanation of social practice (the third dimension of the analysis)

The third dimension is an explanation of the social practice where the discourse is embedded and of which the nurses are a part. This dimension emphasizes how power relations are created through language and its effect on clinical and social practice. The relationship between text, discursive practice, and social practice is elucidated in the Discussion section.

## Results

Eight nurses from two different hospitals working in 24-hour internal medicine wards were interviewed. Both hospitals are located in cities with a large immigrant population, and all respondents had extensive experience with older adult immigrant patients. Table 1 reports the participants’ details.

**Fig. 1:**
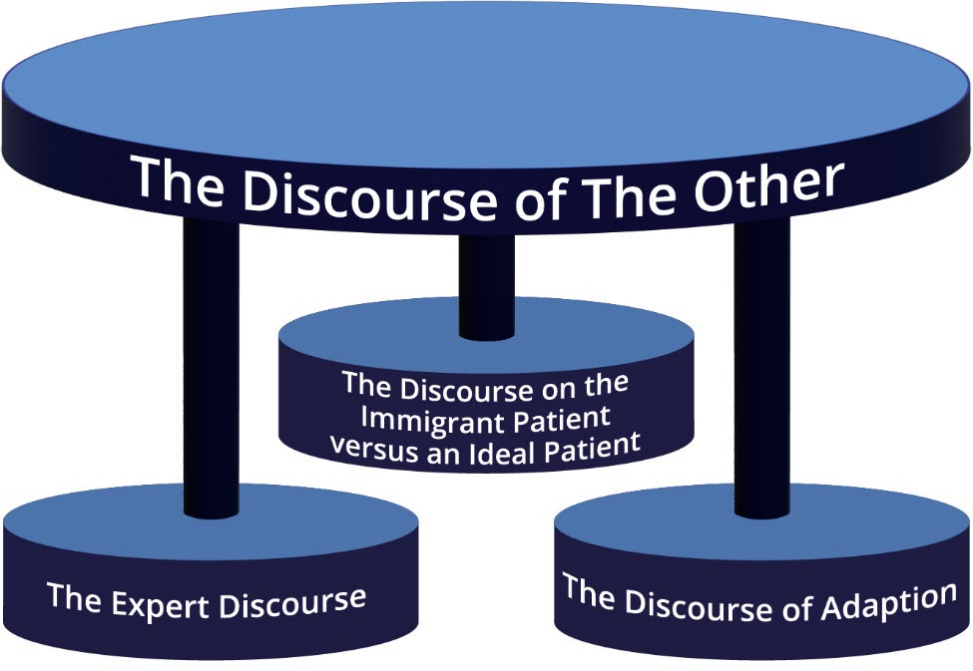
The discursive practice

The analysis of the nurses’ narratives identified an overarching discursive practice, ‘The discourse of the other,’ with three interdiscursive practices as follows: (1) ‘The discourse on the immigrant patient versus an ideal patient’; (2) ‘The expert discourse’; and (3) ‘The discourse of adaption’. The discursive practice, including examples from the linguistic description, is detailed below (Fig. 1).

### The discourse of the other

The nurses stated that immigrants were similar to other patients; however, their narratives included certain linguistic expressions that indicated how older adult immigrants differed from what they expected from other patients. The nurses expressed that they were different, and alien, and *they* were not like *us.* One nurse chose *jungle* as a linguistic image:

Imagine if you’re on holiday in the jungle or somewhere like that, and you end up in a hospital. That’s the sort of hopelessness the patient experiences (Nurse 3).

In Norway, the jungle represents the most foreign and unknown place imaginable. Hence, the nurse indicated that the hospital setting in Norway was as unfamiliar to immigrants as a hospital in the jungle would be to herself. This highlighted the nurse’s perception of older adult immigrants, and the nurse constructed a patient who was considered foreign and without knowledge of the Norwegian health care system.

Older immigrants were rarely referred to as individuals; they were defined as ‘the others’ through the nurses’ use of the plural personal pronouns *we* and *they.* The pronoun *we* was never used to refer to the nurse-patient relationship; the immigrants belonged to one group, *the others*, which was different from *we*, the group to which the nurses belonged. The quote, ‘*They* view illness completely differently than *we* do’ (Nurse 3), reveals the presentation of *we* and *they* as a dichotomy with asymmetric value and mutually exclusive categories.

The nurses categorized the others based on recognizable similarities, such as language skills, family size, or country of origin, and subsequently, attributed stereotypical characteristics to the patients, such as being passive, not adhering to the nurses’ recommendations, and having a different understanding of illness. The construct of the others as ‘non-Norwegian’ was the most noticeable categorization. One nurse mentioned many family members visiting and said: ‘I think we might have reacted if a Norwegian family had taken up so much space’ (Nurse 6), implying that the family was not Norwegian. Additionally, the nurses used words such as *everybody*, *nobody*, *often*, and *rarely*, leading to generalization; the nurses appeared to view and manage ‘the others’ as a homogeneous group.

The discourse of the other revealed how the nurses constructed and defined older adult immigrants as patients who were ‘different’ and alienated; ‘they’ were not like ‘us’. However, the choice of words also indicates that the nurses occasionally felt alienated, especially when many family members visited the patient.

I almost said that I’ll stand by the door then (laughs). You just don’t want to disturb. (Nurse 1); When you enter a room, and there are… (…) … if there are very many people in the room, the communication is sort of… you feel that they are watching what you are doing. In a way, you feel that you are intruding… (Nurse 6).

In this case, the nurses were in the minority, and the choice of words (*disturb, they are watching you*, and *you are intruding*) indicates that the nurses apparently felt uncomfortable, like an intruder, and outside the norm.

### The discourse on the immigrant patient versus an ideal patient

In this discourse, the nurses constructed the older immigrant patient relative to an active and participatory patient. Immigrants did not behave as expected and did not conform to what seemed to be the nurses’ ideal of a normal patient.

The nurses described older adult immigrants as passive patients, who did not always follow the nurses’ advice. Words such as *different, unfamiliar, normal*, *abnormal, and problem* were used.

Because some of it is so far from what I consider normal, so it’s a bit of a problem (Nurse 8). It was for just a few hours… but my goodness, they turned the whole ward on its head, so to speak (Nurse 3).

In this case, the nurse communicated that *some* things about immigrants were *so far* from her assumption of normal. Abnormal is the antonym of normal; this was negatively presented using the word *problem*. *Turning the ward on its head* generates a chaotic image. This is an orientation metaphor wherein the body indicates space, direction, and the normal and sensible. Thus, the nurse expressed that the choices of the patient and their family were contrary to her ideal of the normal.

#### Passive patients who did not always follow the nurses’ advice

The nurses expressed a perceived expectation, from the patient and their families, that the patient needed rest. This was described as follows.

They lie down under the covers and are very sick… they should be on their feet, but they lie down anyway because they are sick (Nurse 3). Very many of them often want us to do everything for them. No, I’m not here to… it isn’t really a hotel. And if they resist completely, I think… well, I’ll just get their food. But, you feel a bit disheartened. You have many other patients, and we are rushing through the corridors here (Nurse 7).

The expressions *lie down* and *on their feet* can be interpreted literally and metaphorically. Metaphorically, *on their feet* indicates resourcefulness, independent thinking, and sensible choices. This quality is positively valued in Norwegian culture. Thus, the metaphor implies that older immigrants did not make sensible choices or know what was good for them. Additionally, these patients were compared to hotel guests. They were unmotivated and not participative, and the statement, *if they resist completely, I think… well, I’ll just go get their food* implies that they did not always follow the nurse’s recommendations. The negative word *disheartened* and the metaphor *we are rushing through the corridors here* reveal the nurse’s opinions.

#### Women versus men

The nurses used words highlighting a gender disparity.

Naturally, there are challenges, because especially older women who have lived in Norway for 20 years… can’t speak a smidgen of Norwegian. And they cling to their culture, I mean… They cling… yes, and they can’t speak a smidgen of Norwegian (Nurse 3).

Repetitive usage of *cling* and *can’t speak a smidgen of Norwegian* highlighted the additional challenges associated with older adult women immigrants. The expression *cling to their culture* creates a distance and contrast between the women’s culture versus the nurse’s culture. The nurses implied that these patients were inflexible and un-adapted to Norwegian culture. However, the issue with women was not just mutual linguistic comprehension; the nurses expressed that these patients were more likely to let others make decisions and not express their own thoughts. A nurse opined:

I rarely feel that older women… I rarely feel that they want to take action and try to communicate (…) I feel that they are a bit secluded (Nurse 6).

Thus, the nurse generalized older adult women immigrants as passive patients by repeating the word *rarely*.

### The expert discourse

The nurses used words, including auxiliary verbs, indicating that they knew the patient’s best interests. This was justified as follows:

In terms of the profession, you (i.e. the nurse) must just be strict, you must just do it… you (i.e. the patient) must understand now. You (the patient) can’t do what you are doing, in a way (…). Sometimes we just have to whip them up, so to speak (Nurse 3).

The word *strict* is often associated with an authority figure, particularly, a parent. This indicates that the patient did not follow the nurse’s orders if she expressed herself more mildly. The modal auxiliary verbs *must* and *can* were repeatedly used. *Must* implies an instruction and a lack of choice for the patient. The nurse’s statement *you can’t do what you are doing*, can be understood as a prohibition wherein the patient was prevented from making their own assessments or choices. This was reinforced through the metaphor *whipping them up*, conventionally associated with slaves, or domesticated animals. The nurse’s expression *we just have to whip them up*, indicated that she alone knew the patient’s best interests and that there were no other options. Another nurse spoke about a patient who sought assistance:

It makes it a bit wrong that they… when I think that you should be able to do this… (…)… it’s hard to accept, that I have to do it this way (Nurse 8).

The word *wrong* referred to the patient’s wish for assistance, implying that the nurse’s understanding would have been more correct. Moreover, if the patient’s actions were contrary to the nurse’s ideal of ‘right’, negatively charged words, such as *hard to accept*, were used.

In the expert discourse, nurses expressed that they knew the patient’s best interests and behaved accordingly. Hence, older adult immigrants were constructed as patients who were unable to assess or comprehend their own best interests, and, had limited opportunity to involve themselves in decision-making.

### The discourse of adaption

The nurses desired to treat immigrants in a caring relationship, even though they differed from the regular patients. The discourse of adaption describes the nurses’ approach to this aspect. Their narratives comprised words such as *a need to adapt, adjust*, and be *creative*. Thus, older adult immigrants were constructed as patients who needed a different approach. Most nurses clarified that they had little or no professional knowledge about older adult immigrants and their need for adaptation. Nonetheless, they rarely discussed these issues with colleagues. An area where the nurses indicated a need to think differently was communication and interaction. This resulted in a ‘creative’ nurse:

You become a kind of… MacGyver nurse… How do I do this, what do I do in this case? (Nurse 5).

The nurse compared herself to the fictional television action-hero MacGyver, who uses creative solutions and simple items to aid him in impossible situations. As for impossible communication, the nurses stated that different approaches were attempted to get the patient to understand and be understood. For example, they simplified their language, avoided irony and humour, spoke more slowly, used fewer words, and expressed themselves using body language, drawing, or Google Translate.

In addition, they allowed adjustments contradictory to professional recommendations. One nurse opined, based on her professional knowledge, that the patient should have been more active; nonetheless, she allowed the patient to remain in bed because: ‘it’s a balancing act. I don’t want to be disrespectful’ (Nurse 2).

#### A limit for adaptation

The nurses expressed that they chose to be *flexible and open* and had to *adapt and adjust.* However, such statements were partially followed by a *but*.

Well, we… we want to be open, *but*… (Nurse 6); Well… I sort of respect it, *but*… (Nurse 7).

The conjunction *but* introduces a break in the context and expresses a limitation and contradiction to the earlier statement. Another nurse spoke about the number of visiting family members and used the term *tolerance limit* (Nurse 6). Tolerance is permitting something one dislikes or disagrees with, and the term *tolerance limit* indicates the limit to which the nurses were willing to endure, in the context of alienness and difference.

Table 2 summarises the discourses and how older adult immigrants were constructed as patients.

**Table 2:** Discourses and how older adult immigrants were constructed as patients

The discourse of the other
• A patient who was considered different, ‘they’ were not like ‘us.’ • A homogeneous group and not an individual person. • A patient with stereotypical characteristics based on recognizable similarities. • A patient who did not know the Norwegian healthcare service.
**The discourse on the immigrant patient versus an ideal patient**
• A patient who did not conform to (what seemed to be) the nurses’ ideal of a normal patient. • A patient in contrast to an active and participatory patient. • A passive patient who did not always follow the nurses’ advice. • A patient who did not make sensible choices.
**The expert discourse**
• A patient unable to assess or comprehend their best interests. • A patient with limited opportunity for involvement in decisions that concern themselves.
**The discourse of adaption**
• A patient who needed a different approach; however, due to alienness and unfamiliarity, there was a tolerance limit for adaption by the nurses.

## Discussion

This study aimed to explore the discursive construction of older immigrant adults as patients in hospitals, by analysing the nurses’ narratives (Fig. 1; Table 2). The ‘discourse of the other’ was the overarching, dominant, and stable discourse, with three interdiscursive practices: (1) ‘The discourse on the immigrant patient versus an ideal patient’ constructed a patient who did not conform to what seemed to be the nurses’ ideal of a normal patient, that is, an active and participatory patient; (2) ‘The expert discourse’ constructed a patient unable to assess or comprehend their own best interests; and (3) ‘The discourse of adaption’ constructed a patient who needed a different approach. Individually and together, the discourses explain some aspects of the social practices nurses and patients are a part of [37]; this will be discussed below.

Fairclough [37] highlights that discourse constructs our perceptions of relationships, identity, power, and knowledge. In the present study, the nurses constructed the patient’s identity as ‘the other’, an identity that contrasts with what seemed to be the nurse’s ideal of a ‘normal’ patient. A notion of normality presupposes that those outside the norm are seen as othered [44]. ‘Othering’ is a term used in nursing literature for the process of relating to those who are perceived to be different from ourselves [45]; however, othering is not limited to individual interactions; structural othering also exists, as individual actors, such as nurses, operate within normative systems, structures, and institutions [46].

Othering is probably not a deliberate act by the nurses; it could be seen as the result of norms that have become naturalized to the extent that nurses simply take them for granted, without reflecting on the consequences for social practice or care delivery. Nurses will, regardless of well-intentioned practice, be influenced by factors, such as societal norms, dominant ideologies, and frameworks for what is perceived as normal in the hospital as an institution. First, the meeting between older adult immigrants and nurses is a meeting between generations, as well as a meeting between the minority and majority societies, respectively. The majority possesses power and can set the premises and define, influence, and identify norms and values that marginalize those who fall outside these norms [47]. Additionally, nurses’ norms are influenced by prevailing public discourses. Immigrants, in a European context, are often subject to a negative portrayal by the media [48], and Nordic governmental documents on immigrants construct them as passive service recipients with little latitude for influence [36]. A study by Arora et al. [18] highlighted this interplay between micro- and macro-contexts and found that a macro-context, such as ongoing negative public discourses about Pakistani immigrants, spilled over and affected the interaction between healthcare professionals and patients as a micro-context.

Othering puts older adult immigrant patients at risk for suboptimal care [49] and influences negative trust-building in patient-healthcare encounters [50]. As health professionals, nurses must treat all patients as unique and equal, though discourses in this study reveal that this is somewhat absent in social practice when it comes to patients who are older adult immigrants. When patients are ‘othered’ by nurses, it reveals a social practice in which nurses contribute to objectification of patients by causing the other person’s unique individuality to disappear [51]. The World Health Organization emphasizes the importance of a person-centred approach to provide equal care [28]; in this study, however, older adult immigrant patients were objectified and constructed without a personal identity, categorized, and subsequently attributed with stereotypical characteristics. According to Canales [45], persons are labelled like this due to perceived differences from the societal norm. The ‘discourse of the other’ thus reveals a social practice where generalization is more prevalent than a person-centred approach in cases of older adult immigrant patients.

Nursing is based on respect for human rights and the humanist ideal that all people are unique, irreplaceable, and have inherent dignity [52]. Conversely, othering, categorization, and generalization lean towards a reductionist view of humanity, where people are not seen as unique holistic individuals. This may apply to patients in general; however, due to the discourses in this study, it may appear that older immigrant patients are particularly at risk.

In the adaption discourse, the nurses expressed that they had to adapt and adjust. Thus far, this is in line with recommendations emphasizing that care shall be adapted to the individual’s background to achieve equality in health services [52]. However, norms can affect the care provided and be an obstacle to equality in health service, whether they are explicit, conscious, or unconscious [44]. The discourse of adaption indicated a social practice where prevailing norms were inadequate for dealing with older adult immigrant patients, and the term tolerance limit reveals that the nurses had the power to decide to what extent they allowed and accepted individual adaptations to older adult immigrants. The power of tolerance was held by the nurses, and the older adult immigrant patients who were tolerated were at the mercy of the nurses’ choices.

In this study, the discourse on the immigrant patient versus an ideal patient points to a social practice where an active and participative patient is considered ideal by the nurses and immigrants are constructed in relation to such a patient. Nurses’ norms regarding patients are influenced by several factors, including the legislative framework. In recent years, the evolving patient role has expected patients to be more involved [28]; patient participation has become an explicit health policy ideal. However, in this study, older adult immigrants were simultaneously constructed as patients unable to judge or know what was in their own best interest. This became apparent in the expert discourse; nurses appeared to be influenced by Parsons’ ‘sick role’, a predominantly paternalistic ideology [26]. Thus, the ‘expert discourse’, reveals a social practice in which paternalism overrides patient autonomy when the patient is an older adult immigrant. This indicates a duality; the nurses emphasized that active and involved patients are ideal, and yet they gave immigrants reduced agency and autonomy.

While participation potentially influences and changes power relations [53], the nurses’ construction in this study indicates a social practice in which older adult immigrant patients have a reduced opportunity to participate and, thus, are not in a position to negotiate for power. However, the narratives contained a few examples where the nurses themselves felt alienated; this was typically seen in situations where the nurse was in the minority and, thus, felt ‘different’ and outside the norm. The nurses constructed themselves as experts; however, in these situations immigrant patients sometimes made them feel uncertain of their professional capabilities. This is one of the few examples in this study where power relations were negotiated, and more power was transferred to the patient. Another example is in the discourse of adaption; the nurses described a lack of cultural knowledge regarding older adult immigrants, and this tallies with findings in other studies [20]. Nevertheless, the nurses challenged their own existing norms to adapt a caring relationship in the best possible way according to the patient’s wishes. This can be seen in line with Foucault’s term ‘Contre-conduite’ [54], a counter-behaviour from the nurses, and a willingness not to allow themselves to be governed or disciplined by prevailing norms. The nurses were present and allowed themselves to be influenced and touched by the patients; they demonstrated a critical reflection where they thought and acted outside the prevailing and established norms.

Nevertheless, the discursive practice in this study points to a social practice where nurses directed more attention to what they perceived as deviations from the norm, rather than reflecting on and questioning the basis for their own norms. This may be because the interests and perspectives of the majority are presumed natural and universal for the nurses.

### Strength and limitations

This study aims to explore discursive constructions; hence, CDA was chosen. Discussion on quality criteria within CDA is limited. Thus, in this study, aspects of credibility, transferability, dependability, and confirmability are used to ensure trustworthiness [55].

A purposive sample based on certain inclusion criteria was used to gain *credibility*. The present study has a social constructivist approach; the nurses’ narratives are constructed in dialogue with the researcher and, hence, cannot be seen as an accurate representation of the real world. The small sample size of eight could be a limitation; however, the study aims to explore how older adult immigrants are discursively constructed as patients by these nurses, at this given time and in this context. The nurses who participated had extensive experience with older adult immigrant patients and were, thus, able to provide detailed narratives; therefore, the sample was considered large enough. The nurses were asked to describe their experiences with older immigrant patients who were born and raised in countries outside Europe, and we trust that the provided narratives were about those patients. However, the nurses comprised only ethnic Norwegian women, although variation and diversity within gender and ethnicity were desirable. Other nurses (e.g., from other units, hospitals, or geographical areas) may have constructed different discourses about older adult immigrant patients.

Regarding *transferability*, we cannot conclude that the findings are transferable to other contexts; however, the authors have tried to provide clear descriptions of context, sample, data collection, analysis, and interpretation process. In addition, choices made throughout the research process are described to strengthen *dependability* and create transparent research. COREQ, a checklist for qualitative studies [56], was used in the preparation of the manuscript, to ensure that important aspects of qualitative research were reported (Additional File 1). Regarding *confirmability*, the authors applied a reflexive approach to all parts of the manuscript as we are co-constructors of the narratives, interpretations, and discourses. The first author, who conducted the interviews, tried to bridle her preunderstanding by adapting a self-reflexive approach. The interviewer’s professional experience as a nurse was both a weakness and a strength. It was a strength because of field-based knowledge and familiarity with the ‘language’ of the nurses, but also a weakness because feedback from the interviewer might have influenced the narratives. The analysis was a collaborative effort by the authors, where reflexivity was central. The researchers’ work experience regarding older adult immigrant patients ranged from little to no experience. However, as beliefs and ideologies are embedded within the researcher, the analysis is inevitably guided by the researcher’s preconceptions. Therefore, the analysis and interpretation of discursive practices have been extensively discussed by the authors to pause, reflect, and challenge personal experiences and preconceptions.

## Conclusion

Older adult immigrants are constructed by nurses as ‘othered’ and different patients. The examples discussed show how the language and discourses of caregivers contribute to a social practice with unequal power relations and how discursive practices can have a major impact in a care context. Discursive practice indicates a social practice where paternalism overrides patient autonomy and generalization is more prevalent than a person-centred approach. Furthermore, discursive practice indicates a social practice where nurses’ norms are the basis for normal. Belonging to the norm confers power; normality is presumed and desirable. Older immigrants as ‘othered’ patients do not conform to the nurses’ norm. This contributes to unequal power relations in an otherwise asymmetrical patient-professional relationship wherein the nurse possesses the power to decide the normal, define the other, and determine the extent of adaptation and tolerance. However, there are a few examples where power relations are negotiated, and more power is transferred to the patient. In the discourse of adaptation, the nurses showed counter-behaviour and challenged their own existing norms to adapt a caring relationship in the best possible way as per the patient’s wishes.

Nevertheless, it can be assumed that older adult immigrant patients have a reduced opportunity to participate, they have restricted agency, and are rather powerless in the care relationship. Hence, the language and discourses used by nurses may pose a threat to equal healthcare delivery.

### Implications for practice

This study is important for nurses at the individual level, nursing managers, and nurse educators. The findings reveal how discourses influence social practice and can be an obstacle to providing equal healthcare delivery to older immigrant adults. Moreover, the findings can raise awareness of attitudes, and hopefully interrupt the forces that promote the process of other-directedness in the nurse-patient interaction.

Finally, the study findings have implications for further research. The analysis in this study is based on interviews with nurses, and it will be of interest to explore the immigrant patient’s perspective. Furthermore, it will be of interest to explore and compare any similarities and differences in the way nurses construct older adults as patients, whether they are ethnic Norwegians or immigrants. Future research could also explore adaptation in the nurse-patient interaction when the patient is an older adult immigrant.

## Electronic supplementary material

Below is the link to the electronic supplementary material.

Supplementary Material 1

## Data Availability

The datasets generated and analysed during the current study are not publicly available due to safeguarding the participants’ confidentiality in accordance with the personal data legislation and the Norwegian Center for Research Data (NSD) but are available from the corresponding author on reasonable request.

## Abbreviations

CDA: Critical Discourse Analysis
N: Nurse
